# Knowledge and practices on diarrheal illness and associated factors in Lebanon

**DOI:** 10.3389/fpubh.2025.1618648

**Published:** 2025-12-02

**Authors:** Camille Akkari, Georges Kerbage, Diana Malaeb, Souheil Hallit, Rabih Hallit

**Affiliations:** 1 School of Medicine and Medical Sciences, Holy Spirit University of Kaslik, Jounieh, Lebanon; 2 College of Pharmacy, Gulf Medical University, Ajman, United Arab Emirates; 3 Applied Science Research Center, Applied Science Private University, Amman, Jordan; 4 Department of Infectious Disease, Bellevue Medical Center, Mansourieh, Lebanon; 5 Department of Infectious Disease, Notre Dame des Secours, University Hospital Center, Byblos, Lebanon

**Keywords:** diarrhea, knowledge, Lebanon, practices, public health

## Abstract

**Objectives:**

This study focuses on diarrheal disease in Leba4non, where it remains a significant contributor to childhood morbidity and mortality. Understanding the population’s knowledge and practices regarding diarrhea is crucial for designing effective public health interventions. This study assessed knowledge and practices regarding diarrheal illness in Lebanon, aiming to identify gaps and guide future interventions.

**Methods:**

A cross-sectional survey was conducted from July to October 2024 using snowball sampling among 455 Lebanese residents aged 18 years and above. Data were collected via a self-administered questionnaire, adapted from validated tools, translated into Arabic, and pilot-tested. The survey covered sociodemographic characteristics, knowledge and practices related to diarrhea. Cronbach’s alpha values for knowledge and practice scales were 0.91 and 0.66, respectively.

**Results:**

Participants had a mean age of 35.3 years, and 56.5% were female. Most (71.9%) had never received information about diarrheal illness. Higher knowledge was observed among medical professionals and university graduates, while lower knowledge was associated with overcrowded households and having more children under five. Better practices were observed among women and those who were previously informed about diarrhea.

**Conclusion:**

The findings of this study revealed that while some participants demonstrated awareness regarding diarrheal illness, with over two-third reporting having no prior information about the disease. These results highlight important knowledge gaps within the Lebanese population that warrant attention. To address these gaps, targeted health education initiatives focusing on diarrhea prevention, management, and care-seeking behaviors are recommended to enhance public awareness and promote better health practices.

## Introduction

According to the World Health Organization (WHO), diarrhea is the third leading cause of death among children aged 1 month to 5 years, defined as either the passage of three or more loose or liquid stools within a 24-h period or an increase in frequency beyond what is normal for the individual (1). It can result in dehydration, electrolyte imbalances, malnutrition, impaired cognitive development, and reduced physical and educational progress (2–4).

Diarrhea has a disproportionately high impact on morbidity, especially in low- and middle-income countries, where limited resources and weaker infrastructure hinder effective health management compared to high-income nations (5–7). Lebanon has been facing a severe economic and health crisis since 2019, which has led to an increase in infectious outbreaks, including diarrheal illnesses. This is largely due to low socioeconomic status (SES), poor living conditions, inadequate water sanitation, and food safety (8, 9). Additionally, the collapse of infrastructure has resulted in unsafe drinking water, poor sanitation, limited hygiene practices, and insufficient access to water treatment options (10). With overcrowded living conditions and limited access to necessities like clean water, electricity, and safe food, many communities are increasingly vulnerable to diarrheal illnesses (10).

While Lebanon has a national action plan to combat antimicrobial resistance, another major issue is the widespread and unregulated use of antibiotics, which are easily accessible in pharmacies without a prescription (11–15). Insufficient surveillance, weak enforcement of regulations, and public misconceptions about antibiotic use have fueled overuse and misuse of these drugs, ultimately leading to the development of antimicrobial resistance (AMR) (15). This resistance makes infections caused by resistant bacterial strains more difficult to treat, posing significant public health challenges. In the case of diarrheal illness, this resistance can lead to more severe and prolonged infections, creating a vicious cycle that heightens public health risks (16). Effective practices against diarrhea focus mainly on preventing dehydration (17) through maintaining the usual diet and replenishing lost fluids and electrolytes (8), continuing breastfeeding (18) and zinc supplementation (19).

Thanks to effective prevention and treatment strategies, mortality rates have dropped to nearly a quarter of their levels from the 1980s to the 2000s (20–22), but the number of deaths caused by diarrhea remains alarmingly high (20–22) and improvements in how diarrhea is managed at home remain limited (23). In many developing countries, such as Lebanon, the ability of caregivers, especially those with young children, to effectively manage diarrhea at home is hindered by gaps in knowledge and awareness (24). In fact, similar studies have been conducted in countries such as Nigeria, Ethiopia, and India (25–30), and have identified significant gaps in both knowledge and practices related to diarrhea management. These studies highlighted that some caregivers often lack understanding of the disease’s symptoms, causes, and prevention methods, affecting their ability to manage it effectively. They also emphasized the crucial role of education in improving practices, with health education programs proving effective in increasing knowledge and promoting better prevention measures like proper hydration, oral rehydration salts (ORS) use, and improved hygiene (19). These findings underline the importance of similar educational interventions in Lebanon to address knowledge gaps and improve diarrhea management practices. Additionally, studies in the MENA region on cholera knowledge, attitudes, and practices have shown that even in outbreak and non-outbreak settings, significant gaps exist in public awareness and management of diarrheal diseases. For instance, lower education levels and residing in cholera-free countries were associated with poorer knowledge, while medical sector participants demonstrated high knowledge that did not always translate into effective practices. These findings highlight the importance of targeted education programs, hygiene promotion, and infrastructure improvement, which are crucial not only for cholera but for managing other diarrheal illnesses, including those prevalent in Lebanon (31).

Given Lebanon’s ongoing crisis and the unique healthcare challenges it presents, including the over-the-counter sale of antibiotics with minimal regulation (32, 33), there is an urgent need for more comprehensive awareness and education on effective diarrhea management. However, nationwide data on the knowledge and practices regarding diarrheal illnesses in Lebanon remain limited. This study builds on previous research in Lebanon by providing an updated, nationwide assessment of knowledge and practices regarding diarrheal illnesses, aiming to identify current gaps and inform targeted public health interventions. It is anticipated that a higher level of knowledge about diarrhea and its management will be positively associated with factors such as higher education, employment in the medical field, and better socioeconomic conditions, which will, in turn, lead to improved management practices.

## Materials and methods

### Study design

This study employed a cross-sectional online survey design and was conducted among a sample of individuals from various regions of Lebanon, including Bekaa, Baalbek/Hermel, Mount Lebanon, Beirut, North/Akkar, and South/Nabatieh. Eligible participants were Lebanese residents aged 18 years or older. Data were gathered through an electronic self-administered questionnaire, distributed between July and October 2024 using a snowball sampling method, a non-probability approach. Initially, the link was shared with a small number of participants, who were then asked to refer others within their network to take part, explaining the snowball sampling technique followed in participants’ recruitment. The survey link was shared via social media platforms such as WhatsApp, Facebook, and Instagram and remained accessible for the entire study period. The survey remained open beyond the calculated minimum sample size to allow additional participants to respond and was closed after a period with no new submissions, resulting in a total of 455 responses. A cover letter outlining the study’s objectives was provided at the beginning of the questionnaire, and the estimated time to complete the survey was approximately 10 min. This study included Lebanese individuals aged 18 years and older, living in either urban or rural areas of Lebanon, who were willing to participate. Those who were non-Lebanese, under 18 years old, or chose not to participate were excluded.

### Ethical aspects

The study was approved by the Ethics Committee at the Notre Dame des Secours University Hospital Center (CR: 2/2024). All participants provided electronic informed consent prior to participation. Before beginning the first part of the questionnaire, participants were required to indicate their consent electronically.

### Study instrument and outcomes

The study questionnaire (Appendix 1) consisted of two main sections. The first section gathered general sociodemographic information, including participants’ age, sex, area of residence, level of education, occupation, marital status, the number of children in the household, and household crowding index (HCI, calculated as the number of people divided by the number of rooms in the home) (24); refers to the socioeconomic status (SES) of the family, with higher HCI values reflecting lower SES. Financial burden was assessed using a single question where participants rated their financial burden on a scale from 1 (lowest) to 10 (highest) (34). The second section of the questionnaire focused on assessing participants’ knowledge and practices related to diarrheal illness, including its definition, causes, consequences, and management, as well as prevention methods and attitudes toward the disease. The questions were adapted from previous studies examining knowledge and practices toward diarrheal diseases (11, 25, 26, 35–38). To ensure accuracy and cultural appropriateness, the translation process followed a standard forward-backward translation procedure: one bilingual expert translated the tool into Arabic, and another independent translator back-translated it into English. Discrepancies were resolved through consensus by a panel of experts (including one public health specialist and one epidemiologist). The final Arabic version was pretested in a pilot study of 30 participants to assess clarity, comprehension and wording, with no adjustments made accordingly. The questionnaire included questions about the definition of diarrhea (6 Items), its causes (10 Items), consequences (10 items), transmission (5 Items), management (5 Items), treatment, and preventive measures (19 items) and spread (7 Items) of diarrheal illnesses, as well as questions on water sources, water hygiene, sanitation practices, and care-seeking behaviors, such as treatment type and healthcare setting. Practice questions included some about water and sanitation (10 items), places the participant goes to in case of suspicion of diarrheal illness (5 items) and treatment (12 items). Each question in the knowledge and practice assessment was scored using a Likert scale, where higher points given to correct knowledge/practice question. Some questions had reversed coding. All questions in the questionnaire were set as mandatory in the Google Forms platform, and only fully completed responses were submitted and included in the analysis; participants who did not complete all items were automatically excluded. The total score was the sum of the points from all questions, with higher scores reflecting better knowledge and practice. The Cronbach’s alpha values were 0.91 for the knowledge scale and 0.66 for the practice scale. According to commonly accepted thresholds, a Cronbach’s alpha of ≥ 0.70 indicates good internal consistency; therefore, the knowledge scale demonstrated excellent reliability. Although the practice scale’s alpha (0.66) was slightly below this threshold, it is considered acceptable for exploratory research, reflecting adequate dependability of the tool in this context.

### Sample size calculation

The sample size was calculated using the G-power software (35), considering an alpha error of 5%, a power of 90%, a minimal model r-square of 5%, and including 12 predictors in the analysis. The minimum required sample size was determined to be 426 participants.

### Statistical analysis

Data analysis was performed using SPSS software version 27. The normality of the distribution of the knowledge and practice scores were confirmed by calculating the skewness (= − 0.082 and 0.100 respectively) and kurtosis (= − 0.038 and 0.562 respectively); skewness and kurtosis values between −1 and +1 are considered acceptable to prove a normal univariate distribution (36). Student’s t-test was used to compare two means, ANOVA test to compare three or more means, and Pearson’s test to correlate two continuous variables. Then, two linear regressions were conducted taking the knowledge and practice scores as the dependent variables and taking the factors that showed a *p* < 0.25 in the bivariate analysis as independent variables. R^2^ values were used to estimate the proportion of variance explained by predictors. Model assumptions were checked via residual plots (linearity, homoscedasticity, normality) and multicollinearity was assessed with variance inflated factor values < 10. Significance was considered for a *p* value < 0.05. Effect sizes were calculated and reported alongside *p*-values to provide a more meaningful interpretation of the results. For mean differences between two groups (t-test), Cohen’s d was used. For ANOVA comparisons across multiple groups, partial eta squared (η^2^) was reported. Effect size values were Interpreted according to conventional benchmarks (Cohen’s d: small = 0.20, medium = 0.50, large = 0.80; partial η^2^: small = 0.01, medium = 0.06, large = 0.14).

## Results

A total of 455 participants completed the questionnaire, with a mean age of 35.2 ± 11.9 years, and 56.5% females. Moreover, 28.1% of them had previously received information about diarrheal illness. The sociodemographic and other characteristics of the participants are presented in Table 1.

**Table 1:** Sociodemographic and other characteristics of participants (*N* = 455).

	*N* (%)
Gender	
Male	198 (43.5%)
Female	257 (56.5%)
Education level	
School level	102 (22.4%)
University level	353 (77.6%)
Marital status	
Single, divorced, widowed	247 (54.3%)
Married	208 (45.7%)
Occupation	
Unemployed	164 (36%)
Non-medical worker	215 (47.3%)
Medical worker	76 (16.7%)
Residence	
Akkar	38 (8.4%)
Baalback	31 (6.8%)
Beirut	68 (14.9%)
Bekaa	38 (8.4%)
Keserwan-Jbeil	57 (12.5%)
Mount Lebanon	68 (14.9%)
Nabatieh	34 (7.5%)
North Lebanon	88 (19.3%)
South Lebanon	33 (7.3%)
Residence area	
Urban	270 (59.3%)
Rural	185 (40.7%)
Number of children under five	
0	306 (67.3%)
1	93 (20.4%)
2	54 (11.9%)
3	2 (0.4%)
Previously received information about diarrheal illness	
No	327 (71.9%)
Yes	128 (28.1%)
Do you suffer from…	
Celiac disease? *Yes*	72 (15.8%)
Hyperthyroidism? *Yes*	47 (10.3%)
Inflammatory bowel disease *Yes*	30 (6.6%)
	Mean ± SD
Age (years)	35.20 ± 11.90
Household crowding index	1.85 ± 0.62
Financial burden	6.32 ± 2.05
Knowledge	190.24 ± 18.64
Practices	97.40 ± 8.37

Supplementary Table 1 represents the results/percentages retrieved from our questionnaire about knowledge of participants regarding diarrheal illness. Participants demonstrated the highest level of knowledge in the Spread (96.2%) and Definition (93.7%) subdomains. Moderate knowledge levels were observed for Causes (89.2%) and Consequences (78.6%), while the lowest scores were recorded for General knowledge (74.3%) and Prevention (71.6%).

Supplementary Table 2 represents the results/percentages retrieved from our questionnaire about practices of participants regarding diarrheal illness. Participants showed excellence performance in the Management in children under five (92.9%) and Places to go (88.8%) subdomains Moderate levels were observed for Treatment (81.0%) and General practices (77.6%), while the lowest score was found in the Water and sanitation subdomain.

The mean knowledge score was 190.24 ± 18.64 out of 243, equivalent to 78% correct answers, indicating relatively high knowledge. However, the mean practice score was 97.40 ± 8.37 out of 150, representing only 51% of the total, highlighting a gap between knowledge and actual management practices.

### Bivariate analyses of factors associated with the knowledge and practice scores

The results of the bivariate analyses are presented in Tables 2, 3. Knowledge was significantly higher among university graduates compared to school level (193.3 vs. 179.6; *p* < 0.001; 95% CI of the difference −17.6; −9.8), in females compared to males (192.4 vs. 187.5; *p* = 0.006; 95% CI of the difference −8.3; −1.4), in single/divorced/widowed participants compared to married ones (193.0 vs. 186.9; *p* < 0.001; 95% CI of the difference 2.7; 9.5), among medical professionals compared to unemployed and non-medical workers (210.0) compared to unemployed (187.0; 95% CI 184.6; 189.4) and non-medical workers (185.7; 95% CI 183.4; 188.1) (*p* < 0.001), in those living in Keserwan-Jbeil (198.9; 95% CI 194.4; 203.3) compared to the other governorates. Furthermore, knowledge scores were also higher in urban residents than rural (194.4 vs. 184.2; *p* < 0.001; 95% CI of the difference 6.8; 13.6), and having previously received information about diarrheal illness compared to those who did not (202.1 vs. 185.6; *p* < 0.001; 95% CI of the difference −20.2; −12.7). In contrast, those living in overcrowded households (*r* = −0.35; *p* < 0.001; 95% CI −0.43; −0.27) or with more children under five (*r* = −0.35; *p* < 0.001; 95% CI −0.43; −0.27) demonstrated significantly lower knowledge levels.

**Table 2:** Bivariate analysis of demographic and educational factors influencing knowledge and practices scores.

	Knowledge			Practices		
	Mean ± SD	*p*	Effect size	Mean ± SD	*p*	Effect size
Gender		**0.006**	0.263		**0.005**	0.265
Male	187.48 ± 18.71			96.15 ± 8.65		
Female	192.35 ± 18.35			98.35 ± 8.04		
Education level		**<0.001**	0.772		0.491	0.077
School level	179.60 ± 17.66			96.89 ± 9.26		
University level	193.31 ± 17.79			97.54 ± 8.10		
Marital status		**<0.001**	0.331		0.224	0.115
Single, divorced, widowed	193.02 ± 19.47			97.83 ± 8.26		
Married	186.92 ± 17.07			96.88 ± 8.49		
Occupation		**<0.001**	0.594		0.090	0.690
Unemployed	186.99 ± 15.66			96.95 ± 8.08		
Non-medical worker	185.73 ± 17.36			97.06 ± 8.11		
Medical worker	209.97 ± 15.30			99.32 ± 9.47		
Residence		**<0.001**	0.173		**0.006**	0.047
Akkar	169.61 ± 15.43			96.24 ± 9.90		
Baalback	186.90 ± 15.03			96.97 ± 7.83		
Beirut	195.00 ± 17.73			97.84 ± 8.56		
Bekaa	187.82 ± 13.77			93.89 ± 5.85		
Keserwan-Jbeil	198.86 ± 16.87			98.67 ± 7.40		
Mount Lebanon	194.63 ± 19.96			100.66 ± 8.54		
Nabatiye	186.24 ± 12.73			95.85 ± 6.34		
North Lebanon	193.24 ± 18.45			96.74 ± 9.18		
South Lebanon	182.24 ± 16.95			96.67 ± 8.02		
Residence area		**<0.001**	0.567		**0.038**	0.199
Urban	194.38 ± 17.79			98.07 ± 8.71		
Rural	184.18 ± 18.24			96.41 ± 7.76		
Previously received information about diarrheal illness		**<0.001**	0.961		**<0.001**	0.370
No	185.61 ± 16.26			96.54 ± 8.16		
Yes	202.06 ± 19.17			99.59 ± 8.53		

Bold numbers indicate significant *p* value.

**Table 3:** Correlations among sociodemographic factors, knowledge, and practices in diarrheal illness management.

	1	2	3	4	5	6
1. Practices	1					
2. Knowledge	0.13**	1				
3. Age	−0.01	−0.03	1			
4. Household overcrowding index	−0.09	−0.35***	−0.03	1		
5. Number of children under five	−0.07	−0.35***	−0.06	0.33***	1	
6. Financial burden	−0.11*	−0.38***	0.08	0.39***	0.35***	1

*p < 0.05; **p < 0.01; ***p < 0.001.

Practice scores, though overall modest, were significantly higher among females compared to males (98.4 vs. 96.2; *p* = 0.005; 95% CI of the difference −3.7; −0.7), among participants living in Mount Lebanon (100.7) compared to other governorates, in urban residents compared to rural ones (98.1 vs. 96.4; *p* = 0.038; 95% CI of the difference 0.1; 3.2), and those who have previously received information about diarrheal illness (99.6 vs. 96.5; *p* < 0.001; 95% CI of the difference −4.8; −1.4). Higher financial burden was significantly associated with lower knowledge (r = −0.38; 95% CI -0.46; −0.30) and practice (*r* = −0.11; 95% CI −0.20; −0.02) scores, while higher knowledge was significantly associated with better practice scores (*r* = 0.13; 95% CI 0.04; 0.22).

The difference in knowledge between school and university levels of education showed a large effect size (*d* = 0.77), while the occupation on knowledge was also large (η^2^ = 0.59). In contrast, gender differences in practices, though statistically significant, reflected small effect sizes (*d* = 0.27), indicating limited practical impact.

### Multivariate analyses

Our multivariate analysis revealed that medical professionals (*β* = 16.6; *p* < 0.001) and university graduates (*β* = 6.5; *p* < 0.001) demonstrated significantly higher knowledge levels about diarrheal illness, while higher household crowding index (*β* = −5.0; *p* < 0.001) and having more children under five (*β* = −4.5; *p* < 0.001) were associated with lower knowledge scores (Table 4, Model 1).

**Table 4:** Multivariable analysis of factors influencing knowledge and practices in diarrheal illness management.

	Unstandardized beta	Standardized beta	*p*	95% confidence interval
Model 1: Linear regression taking the knowledge as the dependent variable (*R*^2^ = 0.389)				
Gender (females vs. males*)	0.54	0.01	0.718	−2.40; 3.49
Residence area (urban vs. rural*)	−1.29	−0.03	0.412	−4.39; 1.80
Household crowding index	−4.98	−0.17	**<0.001**	−7.45; −2.51
Number of children under five	−4.46	−0.17	**<0.001**	−6.81; −2.10
Financial burden	−0.80	−0.09	0.056	−1.61; 0.02
Previously received information about diarrheal illness (yes vs. no*)	3.20	0.08	0.101	−0.63; 7.03
Education level (university vs. secondary or less*)	6.52	0.15	**<0.001**	2.85; 10.20
Marital status (married vs. single*)	3.08	0.08	0.059	−0.12; 6.28
Occupation (Non-medical worker vs. Unemployed*)	−1.23	−0.03	0.462	−4.50; 2.05
Occupation (Medical worker vs. Unemployed*)	16.62	0.33	**<0.001**	12.06; 21.18
Model 2: Linear regression taking the practices as the dependent variable (*R*^2^ = 0.048)				
Gender (females vs. males*)	2.00	0.12	**0.017**	0.35; 3.66
Residence area (urban vs. rural*)	−0.57	−0.03	0.512	−2.27; 1.13
Household overcrowding index	−0.30	−0.02	0.679	−1.70; 1.11
Number of children under five	0.28	0.02	0.682	−1.05; 1.61
Financial burden	−0.03	−0.01	0.907	−0.48; 0.43
Previously received information about diarrheal illness (yes vs. no*)	2.20	0.12	**0.045**	−2.16; 1.40
Marital status (married vs. single*)	−0.38	−0.02	0.674	−0.72; 2.91
Occupation (Non-medical worker vs. Unemployed*)	1.10	0.07	0.235	−0.78; 2.87
Occupation (Medical worker vs. Unemployed*)	0.38	0.02	0.783	−2.32; 3.08
Knowledge	0.03	0.06	0.273	−0.02; 0.08

*Reference group. Bold numbers indicate significant *p* value.

Regarding practices, being female (*β* = 2.0; *p* = 0.017) and having prior exposure to diarrheal illness information (*β* = 2.2; *p* = 0.045) were linked to better practical management of the condition (Table 4, Model 2). The R^2^ values were 0.389 for the knowledge model and 0.048 for the practice model, showing that 38.9 and 4.8% of the variance of the respective outcomes was explained by the independent variables included in each model.

## Discussion

Our study revealed gaps in knowledge and inconsistencies in preventive practices regarding diarrheal illnesses among the Lebanese population. Participants’ knowledge and practices were significantly influenced by education and profession, with medical workers and university graduates demonstrating higher knowledge and better practices. Additionally, poor socioeconomic factors were negatively associated with both knowledge and preventive practices. These associations were supported by moderate to large effect sizes, indicating that the statistical significance observed is accompanied by meaningful real-world implications. Importantly, these associations were not only statistically significant but also showed large effect sizes in several cases (e.g., education and occupation), underscoring their practical importance in shaping diarrheal illness awareness and management. Although significant predictors were identified for knowledge, the practices model (Model 2) showed low explanatory power (R^2^ = 0.048), indicating that the factors included explained only a small portion of the variation in preventive behaviors. This suggests that while statistically significant, the observed relationships have limited explanatory capacity for behavioral outcomes.

### Diarrheal illness knowledge

Our study found that the Lebanese population showed an acceptable average knowledge score of 190.24 ± 18.64, representing approximately 78% of the total possible score of 243. This result is comparable to findings from other studies on the knowledge of mothers and caregivers regarding the home-based management of diarrhea in different countries, though it is higher than most. For instance, a study in Nigeria reported a knowledge rate of 75.1%, which is slightly lower than our findings. However, it is worth noting that our study population differed from the Nigerian study, which focused on mothers and caregivers, not the general population.

In contrast, studies from Iran and Ethiopia showed lower knowledge levels, with rates of 65% in Iran, 56.2% in the Awi zone, 63.6% in Finote Selam, 63.8% in Ginchi, and 67% in Mareka (27, 37–39). While these differences may reflect variations in educational systems and cultural factors, it is also important to consider the differences in the scales used to assess knowledge across studies. Nevertheless, the findings suggest that Lebanon has a relatively high level of awareness compared to other diverse populations. However, although knowledge levels are generally acceptable, some gaps still need to be addressed. Having higher levels of education was associated with greater knowledge about diarrheal illness. Participants with a university education had a mean knowledge score of 193.31 ± 17.79, while those with only school-level education scored 179.60 ± 17.66. These findings align with those of other studies (40, 41), which also reported a similar association. Education appears to play a crucial role in increasing knowledge about diarrheal illnesses, as individuals with higher education levels are more likely to understand the condition, its causes and transmission, and recognize its symptoms. The difference in knowledge between the levels of education was associated with a large effect size, emphasizing the strong association between education and health literacy. Thus, this large effect size confirms that education exerts a substantial practical impact on knowledge acquisition, beyond mean statistical significance.

Furthermore, our results indicated that being a healthcare worker and having previously received information about diarrheal illness were associated with better knowledge. In fact, healthcare workers showed the highest levels of knowledge, with a mean score of 209.97 ± 15.30, compared to 186.99 ± 15.66 for unemployed participants and 185.73 ± 17.36 for non-medical workers. This is expected, as prior exposure to information about diarrhea, whether through direct education or training, as is the case for healthcare workers, likely enhances understanding of the disease (42). This is consistent with the literature, which shows that health workers possess better knowledge of diarrhea compared to the general population; for instance, research in low-income countries found that health workers were more likely to correctly identify the causes and preventive measures for diarrhea. Additionally, community-based health education programs have been shown to be effective in improving caregivers’ knowledge about diarrhea (43). These findings underscore the importance of healthcare training and exposure to health information in improving knowledge and preventing diarrheal diseases. The very large effect size observed for occupation indicates that professional background is one of the strongest predictors of diarrheal illness knowledge, reinforcing the practical relevance of this predictor.

Moreover, our study results indicated that a higher household overcrowding index was linked to lower knowledge, which fits with the broader understanding that overcrowded living conditions can hinder educational outcomes. Previous research has shown that overcrowding limits access to quiet study spaces, technology, and other resources essential for learning (44). Additionally, sharing small living spaces with many people not only affects physical comfort but also impacts mental and emotional well-being (45). These factors can slow cognitive development and affect academic performance. Over time, children in overcrowded households may struggle to achieve the same level of education as their peers from less crowded homes, creating a cycle that deepens educational disadvantages (44). Although statistically significant, this relationship reflects a moderate effect, suggesting a meaningful but not dominant influence of living conditions on knowledge.

Another interesting finding in our study is that a higher number of children under five in a household was significantly associated with lower knowledge. This is concerning, as children in that age group are the most vulnerable to diarrheal illnesses, which are one of the leading causes of death, due to their underdeveloped immune systems and heightened risk of dehydration (1). This is in accordance with other studies; for example, in Nigeria, in Dorayi Quarters and Nasarawa G. R. A., only 75.1 and 78.7% of caregivers, respectively, had good knowledge about the causes of diarrhea (46). Similar findings were observed in communities like Kanuri and Bura in Borno (27). These gaps in knowledge can influence how caregivers manage diarrhea, which can delay proper treatment and worsen health outcomes. Addressing these knowledge gaps is crucial, particularly in households with young children under five.

### Diarrheal illness practices

Preventive practices are crucial in managing diarrheal illness. In this regard, our study found an average practice score of 97.40 ± 8.37, which represents approximately 51% of the total possible score of 150, which is similar to the one found in a study done in Lagos, Nigeria (53.1%) (3). Additionally, we found that sociodemographic factors were associated with diarrhea preventive practices in Lebanon. Consistent with other findings (47), being female, and having previously received information about diarrhea were both associated with better prevention practices. In fact, female participants had a slightly higher practice score of 98.35 ± 8.04, compared to male participants, who had a score of 96.15 ± 8.65. Although the *p* value was significant, the effect size indicates a small to moderate impact, reflecting limited but practically relevant differences between genders. The association with being female is possibly explained by the fact that women, particularly mothers, are more likely to take an active role in managing their children’s health, as children are the primary sufferers of severe diarrhea and its complications. This aligns with findings from other studies, like one conducted in India (48), which showed that maternal education and prior health information were important factors in improving the practice of ORS preparation and usage.

Similarly, just as we found that having previously received information about diarrhea was related to higher knowledge about the disease, we also found that it was associated with better practices. Individuals who have been educated on diarrhea appear more likely to adopt effective preventive measures, such as proper handwashing, ensuring clean drinking water, and the appropriate use of ORS. This suggests that information dissemination plays a critical role in not only raising awareness but also in promoting practical behaviors that can significantly reduce the risk of diarrheal diseases. The moderate effect size for prior information emphasizes its strong practical role in shaping preventive behaviors, supporting targeted educational interventions. Although the effect size for occupation appeared notable, the association between occupation and preventive practices did not reach statistical significance in our analyses. Therefore, this finding should be interpreted with caution, as it requires further investigation in larger and more representative samples. Lastly, within the water and sanitation category, results indicated that piped water supply, artesian wells, and covered springs were the most reliable and sufficient sources, with over 75% rated as clear and functional. In contrast, uncovered springs and non-improved sources performed poorly across all indicators, showing low reliability and poor water quality. Improved sources and water trucks showed moderate performance but lagged behind piped systems. Overall, these findings highlight disparities in water source quality and emphasize the need to improve non-piped and uncovered water sources to ensure safe and sufficient access for all.

Notably, the practices model had a low explanatory power (R^2^ = 0.048), indicating that the included predictors account for only a small portion of the variability in actual practices. This highlights that many other factors likely influence preventive behaviors, including access to ORS and zinc, time constraints, caregiving responsibilities, intra-household decision-making, prevailing social norms, and cues from health services or community programs. These unmeasured determinants may explain why knowledge alone does not consistently translate into effective preventive practices.

The correlation between knowledge and practices (*r* = 0.13) indicates a positive but weak relationship between the two constructs. In real-world terms, having more information does not necessarily translate into proportionally improved behavior; other factors such as accessibility of resources, personal habits and cultural norms might play stronger roles in this context. Thus, while education and awareness campaigns are beneficial, knowledge alone might not be sufficient to ensure effective behavioral changes. Interventions should address motivational, structural and contextual barriers.

### Study strengths and limitations

There are several limitations to consider when interpreting the study results: (1) The use of an online snowball sampling approach may have introduced selection bias, as participants were more likely to be urban, educated, and digitally connected. This may have limited the representativeness of the sample and affected the external validity of the findings. Future studies should consider combining online surveys with on-site data collection in rural and underserved areas or applying stratified sampling techniques to reduce this bias and improve population representativeness. Additionally, the sample, while sufficient, may not have been fully representative, as it skewed toward females and those with a university education. (2) Response bias could have affected the results, particularly in the section addressing practices, where participants may have reported socially desirable behaviors. (3) The cross-sectional nature of the study means it cannot provide insights into changes in knowledge and practices over time. Longitudinal studies in the future would be necessary to explore how these factors evolve and inform ongoing public health interventions. (4) The higher education level in the sample population may skew the data. The significant impact of education level and the high proportion of university-educated participants suggests our sample may overestimate population knowledge and practices. This limitation should be addressed in future studies by ensuring a more representative sample. (5) Furthermore, the low Cronbach’s alpha values observed in some subdomains (e.g., “Definition” and “Spread” in knowledge, and “Management,” “Places to go,” “Treatment,” and “General questions” in practice) should be noted. They suggest that the items within these subdomains may not fully capture a single coherent construct, and results related to them should be interpreted with caution. Future research should validate these subdomains using more reliable or culturally adapted items to ensure robust measurement. (6) This study did not explore how environmental and geographical factors, such as water supply, sanitation facilities, population density, climate conditions, and waste disposal, may shape knowledge and practices regarding diarrheal diseases. Future studies should account for these regional variations to provide a more comprehensive understanding of the issue. (7) Using the Household Crowding Index (HCI) to measure socioeconomic status may be too simple, as SES includes income, education, and work; future studies should consider these additional factors. (8) Lastly, low R^2^ for practices suggests possible omitted-variable bias, meaning some factors influencing preventive behaviors were not captured.

Despite these limitations, the findings of this study are valuable for guiding policymakers and healthcare professionals in identifying gaps in public knowledge and practices concerning diarrheal diseases. By highlighting areas where awareness and practices can be improved, the study offers insights that can enhance responses to diarrheal disease outbreaks and contribute to more effective disease prevention and control strategies. The research included participants from various locations that enhanced the study’s reliability as well as its general applicability. The survey tool demonstrated excellent consistency when measuring participant knowledge through its high reliability score (*α* = 0.91). Multiple linear regression enabled the analysis to identify significant social and demographic elements which influence knowledge and practices.

### Clinical implications

The results of this study have several important implications for clinical practice and public health. A large portion of participants reported self-prescribing antibiotics for diarrhea, even though many cases are viral and self-limiting. This behavior contributes to antimicrobial resistance, highlighting the need for doctors to educate patients on appropriate antibiotic use, emphasizing that most diarrheal illnesses do not require them. Sociodemographic factors such as lower education and overcrowded living conditions were associated with poorer knowledge, suggesting that patient education should be tailored and individualized, offering clear, accessible guidance to underprivileged populations at higher risk of mismanaging diarrhea. Additionally, doctors should promote effective diarrhea management, including proper hydration, use of oral rehydration solutions, and hygiene practices.

## Conclusion

In conclusion, this study shows that while the Lebanese population demonstrates a generally acceptable level of knowledge and practices regarding diarrhea, notable gaps remain. The findings highlight key areas that warrant future public health attention, including bridging the knowledge–practice gap and designing targeted interventions for vulnerable groups, particularly those with lower education levels and higher household crowding. Future programs and policies could focus on strengthening health education, promoting hygiene and water sanitation, and enhancing awareness of effective diarrhea management to further reduce the burden of diarrheal illnesses in Lebanon.

### Recommendations

Based on the findings of this study, we recommend targeted patient education by healthcare providers, particularly for individuals with lower education or living in overcrowded households, to promote appropriate diarrhea management and responsible antibiotic use. Policymakers should implement stricter regulations on over-the-counter antibiotic sales and prescriptions to reduce misuse and curb antimicrobial resistance. Efforts should also prioritize improving access to clean water, proper sanitation, and hygiene facilities, especially in rural and underserved areas. Community-based public health campaigns are essential to raise awareness about diarrhea prevention, including proper hydration, use of oral rehydration solutions, and hygiene practices. Finally, ongoing research and monitoring are recommended to evaluate the effectiveness of interventions and guide future public health strategies, ensuring that improvements in knowledge translate into better practices and reduced diarrheal disease burden.

## Data Availability

The data that support the findings of this study are available from the corresponding author upon reasonable request.
