# Supplementary material for: Knowledge and practices on diarrheal illness and associated factors in Lebanon
Source: Front Public Health. 2025 Dec 2;13:1618648. doi: 10.3389/fpubh.2025.1618648 (PMC12705612; doi:10.3389/fpubh.2025.1618648)
Supplement: Supplementary file 1 [file Data_Sheet_1.docx]

*Questionnaire adapted and modified from previous literature.*

I agree to participate in a research study entitled “**Knowledge and practices in a sample of the Lebanese population regarding diarrheal illness**”. I understand that I will be expected to participate and respond to one survey regarding diarrheal illness as well as answer a few interview questions regarding the same topic, if needed. Yes □ No □

**SOCIODEMOGRAPHIC CHARACTERISTICS**

**Age (in years)**: **……...**

**Gender**: Male 1 □ Female 2 □

**Education**: Primary 1 □ Middle School 2 □ Secondary 3 □ University 4 □

**Marital Status**: Single 1 □ Married 2 □ Divorced 3 □ Widowed 4 □

**Occupation**: Unemployed 1 □ Working in the medical field 2 □ Working outside the medical field 3 □

**Residence**: Akkar 1 □ Baalbek-Hermel 2 □ Beirut 3 □ Beqaa 4 □ Keserwan-Jbeil 5 □ Mount Lebanon 6 □ Nabatiyeh 7 □ North 8 □ South 9 □

**Residence Area**: Urban 1 □ Rural 2 □

**Household crowding index (person/room): ……...**

**Number of children under five in the house: ……...**

**Financial burden**: **1** Lowest Financial Burden (LFB) ; **10** Extreme Financial Burden (EFB)

| 1 | 2 | 3 | 4 | 5 | 6 | 7 | 8 | 9 | 10 |
| --- | --- | --- | --- | --- | --- | --- | --- | --- | --- |

**I had previously received information about diarrheal illness**: Yes 1 □ No 0 □

**KNOWLEDGE**

**Definition of diarrhea**

|  | I strongly disagree | I somewhat disagree | I somewhat agree | I strongly agree |
| --- | --- | --- | --- | --- |
| 1. Passage of loose and watery stools |  |  |  |  |
| 2. Passage of mucoid stools only |  |  |  |  |
| 3. Passage of bloody stools |  |  |  |  |
| 4. Passage of no stools |  |  |  |  |
| 5. Passage of hard stools |  |  |  |  |
| 6. Passage of well‑formed stools |  |  |  |  |

**Item 1:** 1 “for Strongly Disagree” to 4 “Strongly Agree”

**Items 2-6:** 1 “for Strongly Agree” to 4 “Strongly Disagree”

**Causes of diarrhea**

|  | I strongly disagree | I somewhat disagree | I somewhat agree | I strongly agree |
| --- | --- | --- | --- | --- |
| 1. Consuming contaminated food or water can cause diarrhea |  |  |  |  |
| 2. Bacterial infections can lead to diarrhea |  |  |  |  |
| 3. Viral infections can lead to diarrhea |  |  |  |  |
| 4. Parasites infections can lead to diarrhea |  |  |  |  |
| 5. Certain medications, such as antibiotics, can sometimes cause diarrhea as a side effect |  |  |  |  |
| 6. Stress or emotional factors can contribute to the onset of diarrhea |  |  |  |  |
| 7. Poor sanitation and hygiene practices can increase the risk of diarrhea |  |  |  |  |
| 8. Food allergies or intolerances can sometimes cause diarrhea |  |  |  |  |
| 9. Excessive consumption of alcohol can be a cause of diarrhea |  |  |  |  |
| 10. Certain chronic conditions can lead to recurrent episodes of diarrhea |  |  |  |  |

**Items 1-10:** 1 “for Strongly Disagree” to 4 “Strongly Agree”

**Do you suffer from celiac disease?** Yes 1 □ No 0 □

**Do you suffer from hyperthyroidism?** Yes 1 □ No 0 □

**Do you suffer from inflammatory bowel disease?** Yes 1 □ No 0 □

**Consequences of diarrhea**

1. Dehydration is a potential consequence of diarrhea.

|  | 1 | 2 | 3 | 4 | 5 |  |
| --- | --- | --- | --- | --- | --- | --- |
| **I strongly disagree** | □ | □ | □ | □ | □ | **I strongly agree** |

1. Malnutrition can occur because of prolonged diarrhea.

|  | 1 | 2 | 3 | 4 | 5 |  |
| --- | --- | --- | --- | --- | --- | --- |
| **I strongly disagree** | □ | □ | □ | □ | □ | **I strongly agree** |

1. Weight loss can be a consequence of diarrhea.

|  | 1 | 2 | 3 | 4 | 5 |  |
| --- | --- | --- | --- | --- | --- | --- |
| **I strongly disagree** | □ | □ | □ | □ | □ | **I strongly agree** |

1. Electrolyte imbalances can develop because of diarrhea.

|  | 1 | 2 | 3 | 4 | 5 |  |
| --- | --- | --- | --- | --- | --- | --- |
| **I strongly disagree** | □ | □ | □ | □ | □ | **I strongly agree** |

1. Fatigue or weakness can result from diarrhea.

|  | 1 | 2 | 3 | 4 | 5 |  |
| --- | --- | --- | --- | --- | --- | --- |
| **I strongly disagree** | □ | □ | □ | □ | □ | **I strongly agree** |

1. Death can occur because of prolonged diarrhea.

|  | 1 | 2 | 3 | 4 | 5 |  |
| --- | --- | --- | --- | --- | --- | --- |
| **I strongly disagree** | □ | □ | □ | □ | □ | **I strongly agree** |

1. Fever can occur in the setting of a diarrheal illness.

|  | 1 | 2 | 3 | 4 | 5 |  |
| --- | --- | --- | --- | --- | --- | --- |
| **I strongly disagree** | □ | □ | □ | □ | □ | **I strongly agree** |

1. Diarrhea poses a greater risk to infants and young children compared to adults.

|  | 1 | 2 | 3 | 4 | 5 |  |
| --- | --- | --- | --- | --- | --- | --- |
| **I strongly disagree** | □ | □ | □ | □ | □ | **I strongly agree** |

1. Diarrhea poses a greater risk to elderly compared to healthy adults.

|  | 1 | 2 | 3 | 4 | 5 |  |
| --- | --- | --- | --- | --- | --- | --- |
| **I strongly disagree** | □ | □ | □ | □ | □ | **I strongly agree** |

1. Diarrhea poses a greater risk to pregnant women and their unborn babies.

|  | 1 | 2 | 3 | 4 | 5 |  |
| --- | --- | --- | --- | --- | --- | --- |
| **I strongly disagree** | □ | □ | □ | □ | □ | **I strongly agree** |

**Attitudes regarding diarrheal illness**

|  | I strongly disagree | I somewhat disagree | I somewhat agree | I strongly agree |
| --- | --- | --- | --- | --- |
| 1. Diarrhea could be contagious |  |  |  |  |
| 2. The spread of diarrheal illness can be stopped |  |  |  |  |
| 3. Travel to infected areas can cause infection |  |  |  |  |
| 4. Can be caused by animal feces |  |  |  |  |
| 5. Vaccines can limit diarrheal illness |  |  |  |  |

**Items 1-5:** 1 “for Strongly Disagree” to 4 “Strongly Agree”

**Prevention of diarrheal illness**

|  | I strongly disagree | I somewhat disagree | I somewhat agree | I strongly agree |
| --- | --- | --- | --- | --- |
| 1. Cannot be prevented |  |  |  |  |
| 2. Herbs |  |  |  |  |
| 3. Washing hands |  |  |  |  |
| 4. Cooking food well |  |  |  |  |
| 5. Heat stored food |  |  |  |  |
| 6. Cover food |  |  |  |  |
| 7. Boil water |  |  |  |  |
| 8. Wash fruits and legumes well |  |  |  |  |
| 9. Clean dishes |  |  |  |  |
| 10. Use toilets properly |  |  |  |  |
| 11. Increase fluid intake |  |  |  |  |
| 12. Decrease fluid intake |  |  |  |  |
| 13. Increase food intake |  |  |  |  |
| 14. Decrease food intake |  |  |  |  |
| 15. Oral rehydrating solution (ORS) use |  |  |  |  |
| 16. Eat more salt and sugar |  |  |  |  |
| 17. Take antibiotics tablets |  |  |  |  |
| 18. Intramuscular drugs |  |  |  |  |
| 19. Herbal medications |  |  |  |  |

**Items 3-4-5-6-7-8-9-10-11-13-15:** 1 “for Strongly Disagree” to 4 “Strongly Agree”

**Items 1-2-12-14-16-17-18-19:** 1 “for Strongly Agree” to 4 “Strongly Disagree”

**Spread of diarrheal illness**

|  | I strongly disagree | I somewhat disagree | I somewhat agree | I strongly agree |
| --- | --- | --- | --- | --- |
| 1. Contaminated water |  |  |  |  |
| 2. Contaminated food |  |  |  |  |
| 3. Bugs |  |  |  |  |
| 4. Bad hygiene |  |  |  |  |
| 5. Poor sanitation |  |  |  |  |
| 6. Kissing |  |  |  |  |
| 7. Person-to-person contact |  |  |  |  |

**Items 1-7:** 1 “for Strongly Disagree” to 4 “Strongly Agree”

| 1. Children lose valuable fluids, salts, and sugars, which can cause shock to vital organs | Yes 1 □ | No 0 □ |
| --- | --- | --- |
| 1. Do you know what oral rehydration therapy (ORT) is used for? | Yes 1 □ | No 0 □ |

**PRACTICES**

**Management of diarrhea in children under five**

|  | I strongly disagree | I somewhat disagree | I somewhat agree | I strongly agree |
| --- | --- | --- | --- | --- |
| 1. Diarrhea is preventable disease and is manageable at home |  |  |  |  |
| 2. Oral rehydration fluids are the first-line treatment of diarrhea in children |  |  |  |  |
| 3. Caregivers can prepare oral rehydration at home |  |  |  |  |
| 4. Giving Oral rehydration fluids at home can treat diarrhea |  |  |  |  |
| 5. Oral rehydration fluids replace the fluids lost in diarrhea |  |  |  |  |

**Items 1-3,5:** 1 “for Strongly Disagree” to 4 “Strongly Agree”

**Item 4:** 1 “for Strongly Agree” to 4 “Strongly Disagree”

**Water and Sanitation Survey – Modified (From Arabic)**

| What is the main source of drinking water? Specify the top three most used sources | | | | |
| --- | --- | --- | --- | --- |
| Water Quality  **Clear 1 /Muddy 2 /Odorous 3** | Requirements  **Sufficient 1 /insufficient 2** | Operational Status  **Good 1 /Not Maintained 2 /Not Working 3 / Don’t Know 4** | Reliability  **Reliable 1 / Unreliable 2** | Drinking Water Source |
|  |  |  |  | 1. Water pipe supply |
|  |  |  |  | 1. Artesian wells |
|  |  |  |  | 1. Shallow wells/Protected springs (covered) |
|  |  |  |  | 1. Shallow wells/Unprotected springs (uncovered) |
|  |  |  |  | 1. Water trucks |
|  |  |  |  | 1. Improved other sources |
|  |  |  |  | 1. Non-improved other sources |
| Are there concerns about the change in water taste? | | | No 0 | Yes 1 |
|  | | | | |
| Have any cases of illness (diarrhea) related to drinking water been reported in the past two weeks? | | | No 0 | Yes 1 |
| Do you treat or purify water to ensure you drink healthy and safe water? | | | No 0 | Yes 1 |

**Place you go if you suspect diarrheal illness**

|  | I strongly disagree | I somewhat disagree | I somewhat agree | I strongly agree |
| --- | --- | --- | --- | --- |
| 1. Hospital |  |  |  |  |
| 2. Dispensary |  |  |  |  |
| 3. Pharmacy |  |  |  |  |
| 4. Herbal shop |  |  |  |  |
| 5. Family/neighbor |  |  |  |  |

**Items 1-3:** 1 “for Strongly Disagree” to 4 “Strongly Agree”

**Items 4,5:** 1 “for Strongly Agree” to 4 “Strongly Disagree”

**Treatment of diarrhea**

|  | I strongly disagree | I somewhat disagree | I somewhat agree | I strongly agree |
| --- | --- | --- | --- | --- |
| 1. Oral antibiotics (Azithromycin, Ciprofloxacin, Metronidazole, etc.) |  |  |  |  |
| 1. Oral antivirals |  |  |  |  |
| 1. Oral anti-diarrheal (Loperamide, etc.) |  |  |  |  |
| 1. Oral rehydration therapy |  |  |  |  |
| 1. Zinc |  |  |  |  |
| 1. Paracetamol |  |  |  |  |

**Items 4,5:** 1 “for Strongly Disagree” to 4 “Strongly Agree”

**Items 1-3,6:** 1 “for Strongly Agree” to 4 “Strongly Disagree”

| 1. The child must receive increased fluids, ORS, zinc, and regular feeding | Yes 1 □ | No 0 □ |
| --- | --- | --- |
| 1. The child requires ORS, but should receive less food in order to reduce the diarrhea | Yes 1 □ | No 0 □ |
| 1. The child should immediately receive antibiotics to stop the diarrhea | Yes 1 □ | No 0 □ |
| 1. Have you ever used oral rehydration therapy (ORT)? | Yes 1 □ | No 0 □ |
| 1. Do you know how to prepare oral rehydration therapy (ORT) at home? | Yes 1 □ | No 0 □ |
| 1. Should the mother continue breastfeeding if her child has diarrhea? | Yes 1 □ | No 0 □ |

[1] Malaeb D, Sallam M, Younes S, Mourad N, Sarray El Dine A, Obeid S, Hallit S, Hallit R. Knowledge, Attitude, and Practice in a Sample of the Lebanese Population Regarding Cholera. Int J Environ Res Public Health. 2022 Dec 4;19(23):16243. doi: 10.3390/ijerph192316243. PMID: 36498316; PMCID: PMC9735709.

[2] Orimbo EO, Oyugi E, Dulacha D, Obonyo M, Hussein A, Githuku J, Owiny M, Gura Z. Knowledge, attitude and practices on cholera in an arid county, Kenya, 2018: A mixed-methods approach. PLoS One. 2020 Feb 26;15(2):e0229437. doi: 10.1371/journal.pone.0229437. PMID: 32101587; PMCID: PMC7043758.

[3] Workie HM, Sharifabdilahi AS, Addis EM. Mothers' knowledge, attitude and practice towards the prevention and home-based management of diarrheal disease among under-five children in Diredawa, Eastern Ethiopia, 2016: a cross-sectional study. BMC Pediatr. 2018 Nov 19;18(1):358. doi: 10.1186/s12887-018-1321-6. PMID: 30453926; PMCID: PMC6241041.

[4] Omole, V., Wamyil-Mshelia, T., Aliyu-Zubair, R., Audu, O., Gobir, A., & Nwankwo, B. (2019). Knowledge and prevalence of diarrheal disease in a suburban community in north western Nigeria. Sahel Medical Journal, 22(3), 114. https://doi.org/10.4103/smj.smj_50_18

[5] Diarrhoeal disease. (2024, March 7). <https://www.who.int/news-room/fact-sheets/detail/diarrhoeal-disease>

[6] Sesay, B. P., Hakizimana, J. L., Elduma, A. H., & Gebru, G. N. (2023, June 13). Knowledge and practices of the adult population on diarrheal diseases, transmission, and prevention in Sierra Leone: A community-based cluster survey. African Journal of Health Sciences, 36(2), 113–123. <https://doi.org/10.4314/ajhs.v36i2.3>

[7] Nutrition and Water, Sanitation, and Hygiene (WASH) Assessment – Arabic

[8] Alghadeer S, Syed W, Alhossan A, Alrabiah Z, Babelghaith SD, Al Arifi MN, Alwhaibi A. Assessment of Saudi Mother's Knowledge and Attitudes towards Childhood Diarrhea and Its Management. Int J Environ Res Public Health. 2021 Apr 9;18(8):3982. doi: 10.3390/ijerph18083982. PMID: 33918950; PMCID: PMC8069305.

[9] Diarrhoea - IMCI Distance Learning Course, Module 4 - WHO 2014
